# Chemical defense responses of upland cotton, *Gossypium hirsutum* L. to physical wounding

**DOI:** 10.1002/pld3.141

**Published:** 2019-05-17

**Authors:** Sang‐Hyuck Park, Jodi Scheffler, Brian Scheffler, Charles L. Cantrell, Christopher S. Pauli

**Affiliations:** ^1^ Department of Biology Colorado State University‐Pueblo Pueblo Colorado; ^2^ Agricultural Research Service Crop Genetics Research Unit USDA Stoneville Mississippi; ^3^ Agricultural Research Service Genomics and Bioinformatics Research Unit USDA Stoneville Mississippi; ^4^ Agricultural Research Service Natural Products Utilization Research Unit USDA, University Mississippi

**Keywords:** (+) and (−)‐gossypol, *Gossypium hirsutum* L., heliocides, hemigossypolone, nectar, upland cotton

## Abstract

Upland cotton (*Gossypium hirsutum* L.) produces terpenoid aldehydes (TAs) that protect the plant from microbial and insect infestations. Foliar TAs include plus (+)‐ and minus (−)‐gossypol, hemigossypolone, and heliocides. To examine foliar TAs’ response to physical wounding, the four TA derivatives of a fully glanded *G. hirsutum* variety JACO GL were quantified by ultra‐high performance liquid chromatography. The results show that foliar heliocides increased by 1.7‐fold in younger leaves after wounding. While the hemigossypolone level was not affected by the physical wounding, the level of heliocides was significantly increased up to 1.8‐fold in the younger leaves. Upland cotton accumulates concentrated carbohydrates, amino acids, and fatty acids in foliar extrafloral nectar (EFN) to serve as a nutrient resource, which attracts both beneficial insects and damaging pests. To better understand the nectar physiology, particularly to determine the temporal dynamics of EFN metabolites in response to the wounding, a gas chromatograph‐mass spectrometer (GC‐MS) was used to perform metabolic profiling analyses of a *G. hirsutum* variety Deltapine 383 that has fully developed extrafloral nectaries. A total of 301 compounds were monitored, specifically 75 primary metabolites, two secondary metabolites and 224 unidentified compounds. The physical wounding treatment changed the EFN composition and lowered overall production. The accumulation of 30 metabolites was altered in response to the wounding treatment and threonic acid levels increased consistently. GC‐MS combined with Kovat's analysis enabled identification of EFN secondary metabolites including furfuryl alcohol and 5‐hyrdomethoxyfurfural, which both have antioxidant and antimicrobial properties that may protect the nectar against microbial pathogens. This study provides new insights into the wounding response of cotton plants in terms of cotton metabolites found in leaf glands and extrafloral nectar as well as highlighting some protective functions of secondary metabolites produced in foliar glands and extrafloral nectaries.

## 1 INTRODUCTION

Cotton is a major fiber crop and also an important source of oil and protein. The genus *Gossypium* includes 45 diploid (2n = 2× = 26) and seven tetraploid species (2n = 4× = 52); however, due to the superior fiber properties of the tetraploids, upland cotton (*Gossypium hirsutum* L.) has become the major type grown and accounts for 98% of cotton production in the US and worldwide (USDA Agricultural Outlook Forum Cotton, 2018). Conventional breeding efforts have resulted in new cultivars with improved traits, while molecular breeding research has provided new tools for more efficient trait selection, and the recently completed tetraploid genome sequence is expected to provide new avenues to further improve cotton plant (Li et al., 2015; Zhang et al., 2015).

A continuing threat to cotton productivity is insect pests, including *Lepidopteran* species. In the US, a total of 20 million bales were produced in 2014 with significant pre‐harvest losses, including 880,729 bales (4.4%) lost due to the infestation of lygus insects (333,329 bales), thrips (150,479 bales), bollworm/budworm (140,041 bales), stink bugs (130,905 bales), and cotton fleahoppers (37,836 bales). Insects cause not only decreased yields, but also incur economic costs of insecticide treatment ($2.28/acre). Decreasing pesticide use through improving the plant's ability to protect itself, termed host plant resistance, has become a critical component of sustainable cotton production (Williams, 2014).

**Figure 1:**
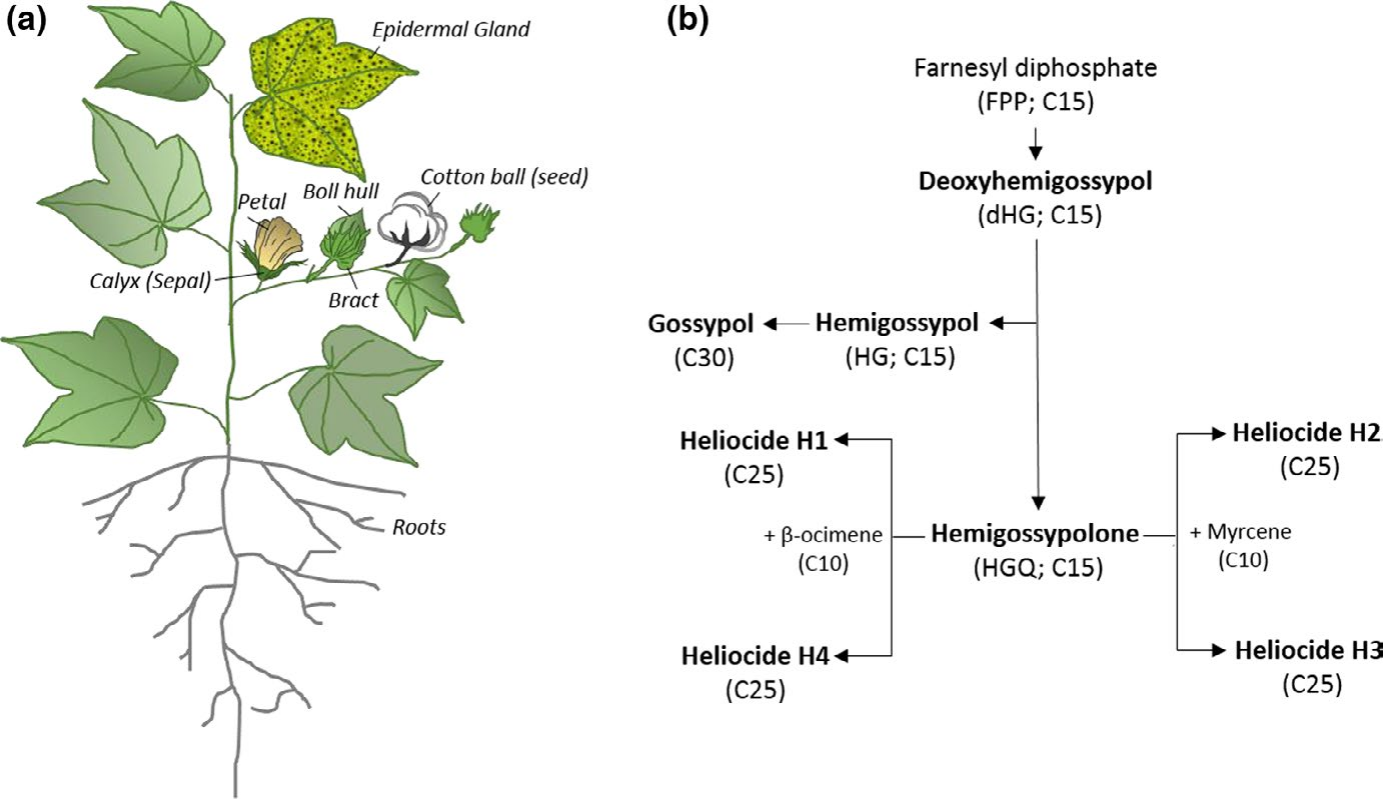
(a) Upland cotton plant and its different parts including a leaf epidermal gland (depicted as a black spot in the leaf) that produces terpenoid aldehyde (TA). (b) TA biosynthetic pathway in the genus *Gossypium*. TAs are derived from the common precursor, deoxyhemigossypol (dHG; C15). The dHG is converted into hemigossypol (HG), then two HG are joined to form gossypol (C30). Hemigossypolone (HGQ) is an oxidized form of dHG that is a precursor to form the heliocides (H1‐H4) (Figure 1b) (Opitz et al., 2008). The number of carbons and precursors are labeled in parenthesis at each step for this catalytic process

Cotton has sub‐epidermal pigment glands that contain a variety of terpenoid aldehyde (TAs) compounds that confer resistance to microbes, viruses, and insects. These glands are found in most parts of the plant, including the roots and seeds (Figure 1a). The TAs found in the glands include the plus and minus isomers of gossypol [(+) ‐ and (−)‐gossypol], hemigossypolone (HGQ), and four heliocide derivatives designated H1 to H4. The amount of each TA present is dependent upon tissue type, age, and environmental conditions. Gossypol is preferentially accumulated in seeds; whereas, HGQ and the heliocides are more prevalent in foliar tissues (Benbouza, Lognay, Scheffler, Baudoin, & Mergeai, 2009). The constitutive and induced gland expression of these secondary metabolites enhances the cotton plants’ self‐protection mechanisms against noctuid caterpillars (Agrawal & Karban, 2000). The systemic induction of TAs was also reported in cotton pigment glands after beet armyworm larvae, *Spodoptera exigua* feedings (McAuslane & Alborn, 1998; McAuslane, Alborn, & Toth, 1997).

Cotton nectaries produce highly concentrated carbohydrates, nectarins (nectary proteins), amino acids, and fatty acids (Anton, Komon‐Janczara, & Denisow, 2017; Heil, 2011). Nectar is an important nutrient source for insects and pathogens due to its high sugar content, which accounts for up to 34% of the total nectar volume in cotton (Chalcoff, Aizen, & Galetto, 2006; Knopper, Dan, Reisig, Johnson, & Bowers, 2016; Nicolson, 2007). Cotton nectar attracts insects beneficial for pollination and protection, which enable the plants to achieve greater reproduction (Gonzalez‐Teuber, Silva Bueno, Heil, & Boland, 2012). By concentrating nectar sucrose, two plants, *Acacia* and *Senna mexicana* var. *chapmanii,* can also strengthen their indirect defense mechanisms through attracting of defending ants (Gonzalez‐Teuber et al., 2012; Jones & Koptur, 2015).

Plants with the nectariless trait (no nectaries) have attracted attention as a biocontrol agent of pests and disease due to an apparent reduction in insect pest damage compared to cotton plants with nectaries (Stenberg, Heil, Ahman, & Bjorkman, 2015). One study showed that the survival, oviposition, and population rate of lygus insects (*L. hesperus* Knight) on nectariless cotton appeared to be lower than a nectaried cotton variety (Benedict, Leigh, Hyer, & Wynholds, 1981). Utilizing nectariless cotton varieties may hold promise to make the crop safer and more profitable by reducing the number of insecticide applications and allowing less toxic insecticides to be used. After years of breeding efforts, the nectariless trait from the wild‐species, *G. tomentosum* was transferred to upland cotton (Meyer & Meyer, 1961). The question remains whether the benefits of removing the nectaries, outweigh the benefits nectar provides to the cotton plant.

Extrafloral nectar (EFN) has traditionally been viewed as an indirect plant defensive mechanism while floral nectar (FN) is considered a part of its reproductive system (Wackers & Bonifay, 2004). The production of EFN is systemically inducible by diverse stimuli, such as herbivore physical damage and phytohormones (*e.g*., jasmonic acid; JA). Studies have showed that in upland cotton the level of carbohydrates increased up to 12‐fold in response to herbivore damage. The elevated sugar level facilitated the plants ability to preferentially recruit predators and parasitoids to the damaged sites and younger leaf tissues (Rudgers, Hodgen, & White, 2003; Wackers, Zuber, Wunderlin, & Keller, 2001; Wagner, 1997).

De la Barrera and Nobel (2004) reported the energy allocation required for nectar production was typically high and appeared to vary among plant species, ranging from 3% to 35%. (De la Barrera & Nobel, 2004). The volume and production of nectar appear to correlate to environmental changes. For example, acorn squash (*Cucurbita pepo*) and *Agave* sp. had peak nectar production when pollen was most available and in cactus (*Stenocereus stellatus*) peak production was observed when their stigmas were most receptive (Casas, Valiente‐Banuet, Rojas‐Martinez, & Davila, 1999; Molina‐Freaner & Eguiarte, 2003; Nepi, Pacini, & Willemse, 1996). Another study reported that unconsumed nectar can be reabsorbed with the nectar constituents recycled. In upland cotton, nectar was shown to be reabsorbed, but at a faster rate in floral nectaries than EFN (Cardoso‐Gustavson & Davis, 2015).

While there has been limited research on secondary metabolites in EFN solutes, several compounds have been identified (Gilliam, Mccaughey, & Moffett, 1981; Hanny & Elmore, 1974; Stone, Thompson, & Pitre, 1985). Due to the lack of chemical catalogs, the understanding of EFN‐associated defense mechanisms remain incomplete. Since nectaries serve as an entry site for a number of microbes including yeast and fungi, it is reasonable for a protective mechanism has evolved against them chemically and physically. The FN of other plant species such as *Nicotiana* spp., *Catalpa speciose*, and *Gelsemium sempervirens* produce a variety of pathogenesis‐related metabolites and protective enzymes such as nectarin, catalpol, and gelsemine, respectively (Heil, 2011); however, no secondary metabolites associated with microbial defense have yet been reported in cotton EFN.

In this research, we use upland cotton plants to investigate how defense compounds such as terpenoid aldehydes (TAs) responded to physical wounding treatments. Extrafloral nectaries following physical wounding were also evaluated for the presence of primary metabolic compounds known to be involved in stress signaling and defense response, as well as other secondary metabolites involved in defense systems.

## 2 MATERIALS AND METHODS

### 2.1 Cotton plant and sample preparation for terpenoid aldehyde (TA) analysis

**Figure 2:**
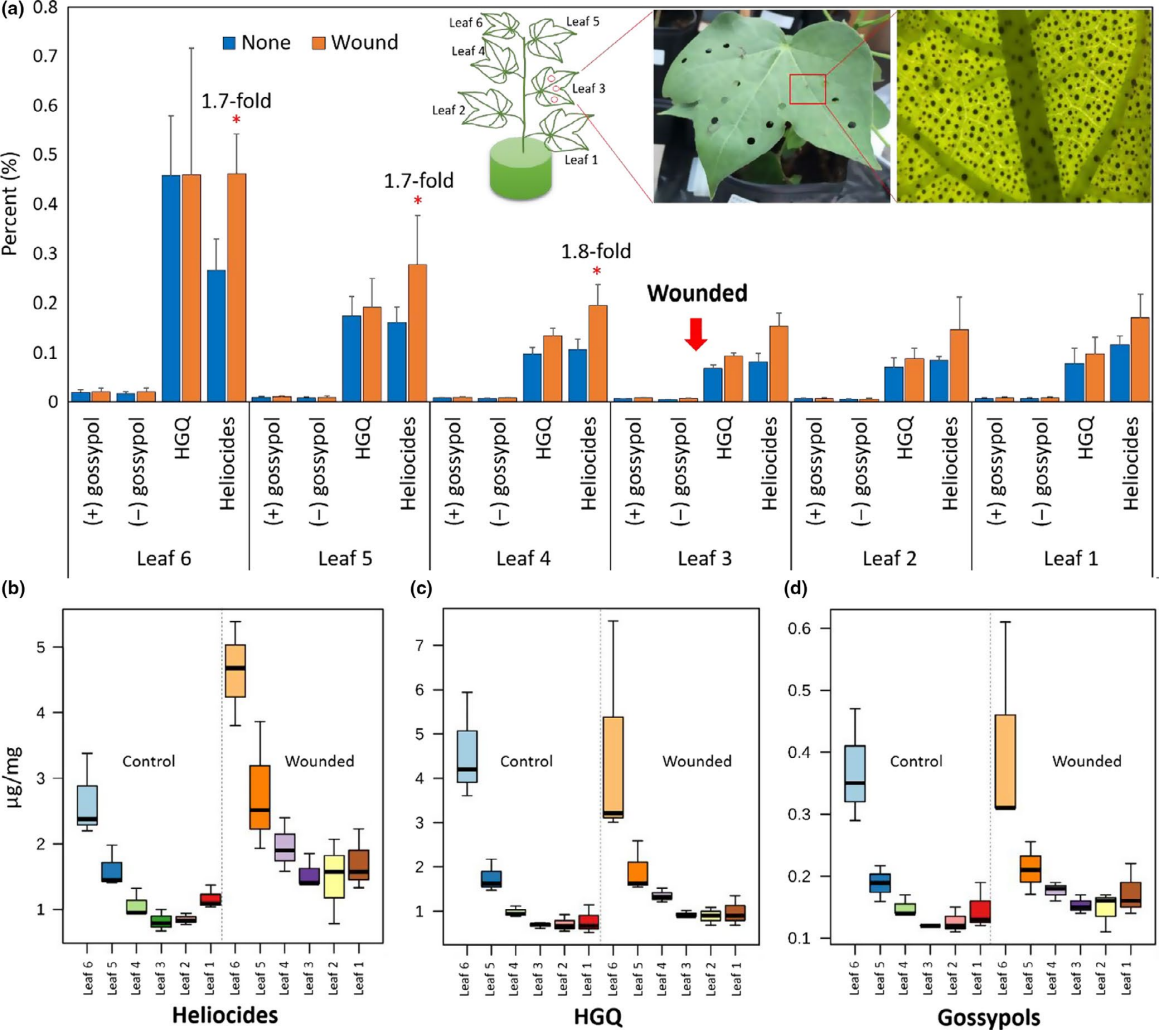
Terpenoid aldehyde (TAs) analysis of JACO GL foliar glands. (a) Different TA levels (% in total mg) in the leaf tissues (leaf 1–6) in response to the physical damage. The insets display a cotton plant in which leaves are numbered (left), a wounded plant at the fourth leaf of cotton using a 1/4ʺ round hole punch puncher hole (middle), and an enlarged image (right) displaying the leaf glands that produce TAs. B. Leaf‐by‐leaf analysis of heliocides (b), HGQ (c), and Gossypol (d) between control and treated cotton plants (One‐way ANOVA and Fisher's LSD as a post hoc analysis, *n* = 3, *p *<* *0.005)

For TA analysis, a *G. hirsutum* variety, fully glanded JACO GL, was grown in triplicate in a growth chamber maintained at 25°C under a 16 hr light/8 hr dark cycle with a 70%–80% humidity level. JACO GL consistently produces TAs in all above ground parts of the plant and is not as affected by environmental variation as some other cotton lines. The 8‐week‐old cotton plants with five or six leaves were subjected to a physical wounding treatment. The treatment was performed on the third leaf up from the bottom of the plant. Fifteen holes were made on the third leaf using a 1/4ʺ round hole punch on two consecutive days. Four days after the first treatment, leaf samples from the top (youngest leaf) to sixth oldest leaf were collected for TA analysis (Figure 2). The leaf samples were stored at −20°C until analysis.

### 2.2 TA analysis using ultra‐high performance liquid chromatography (UHPLC)

Leaves from each position were collected from three control plants and three wounded plants. The leaf samples were freeze‐dried and ground. Samples were extracted using a modified version of the method previously described (Stipanovic, Lopez, Dowd, Puckhaber, & Duke, 2006). Briefly, the extraction consisted of 100 mg ground tissue per 3 ml complexing reagent where 3 ml of the complexing reagent was added (2:10:88, R‐(−) 2‐amino‐1‐propanol: acetic acid: acetonitrile). The sample was heated on a 70°C heat block for 30 min, cooled to room temperature, and vortexed for 30 s. An aliquot was centrifuged at 2,415 ***g*** for 2 min to pellet impurities and the supernatant was diluted 3‐fold with mobile phase (43:37:20, acetonitrile: methanol: 10 mM potassium phosphate pH3) before injection. The diluted sample was poured into an UPLC vial for quantification by UPLC. The UPLC analysis was performed on a Waters Acquity UPLC coupled to a Waters Photodiode Array Detector (PDA) set at 272 nm with scanning from 200 to 500 nm (Waters Corporation). A 2 μl injection was made on a Waters Acquity UPLC HSS C18 column (1.8 μm, 2.1 mm × 100 mm i.d.) connected to a Waters Acquity UPLC HSS C18 VanGuard pre‐column (2.1 mm × 5 mm) with the flow rate set to 0.8 ml/min for 3 min with isocratic conditions (acetonitrile:methanol:10 mM potassium phosphate pH3 [43:37:20]). Retention times in minutes for the TAs were HGQ (0.5), (+) Gossypol (1.1), Heliocides H1 + H4 (1.50 to 1.84) and (−) Gossypol (2.0). Values were estimated using regression to calculate standard curves derived using a range of known concentrations of purified (+) or (−) gossypol, HGQ, or Heliocides H1 to H4. The percentage TA was calculated using the formula % = mg/ml × volume sample (ml)/mg sample × 100. Statistical analyses were performed on normalized data using MetaboAnalyst 3.0 (Xia, Sinelnikov, Han, & Wishart, 2015).

### 2.3 Cotton extrafloral nectar (EFN) sampling

For EFN compositional analysis, the upland cotton variety Deltapine 383 was planted under three environmental conditions in a growth chamber (10–36°C, 17%–100% humidity), a greenhouse (25°C/40°C day/night), and a field near Stoneville, MS. Deltapine 383 is an older variety (PVP 8200137) that does not contain any genetically modified (GMO) traits and has fully developed extrafloral nectaries.

The growth chamber, greenhouse, and field experiments each had 20 plants in triplicate. To avoid possible cross‐talk between plants, the wounded plants were grown in the isolated locations. The 8‐week‐old cotton plants that produced five to six leaves were used for EFN collection. Prior to physical wounding, all previously formed nectar was removed. The wounding was subsequently introduced during days 1 and 2 as described above. At day 4, the EFN samples (0.3–6.2 μl/leaf) were collected at 9 a.m. using a 5‐cm glass capillary microtube. In this study, three biological replicates of untreated control and wounded plants were used for EFN sampling. Each replicate included 20 plants. The average of each replicate was estimated and calculated for total production under each condition. The collected nectars were used for metabolite analyses.

### 2.4 EFN primary metabolite analysis by GC‐time‐of‐flight mass spectrometry

A defined volume (1.5‐10 μl, depending on availability) of EFN was suspended in 500 μl of a solvent mixture containing methanol, chloroform, and water at a ratio of 5:2:2 (v/v). After adding 1.5 μg of the surrogate standard ribitol, the material was extracted by sonication for 15 min in Branson 450 sonication bath (ThermoFisher Scientific) and shook for 20 min at 35°C and 151 ***g*** using Eppendorf Thermomixer (USA Scientific Inc). The extracts were then centrifuged for 10 min at 9,660 ***g***, and the supernatants transferred into a new vial and dried. Dry residues were suspended in 10 μl O‐methoxylamine hydrochloride (40 mg/ml in pyridine, both from Sigma‐Aldrich) and incubated for 90 min at 30°C and 67 ***g***. Subsequently, samples were derivatized with 90 μl of MSTFA with 1% TMCS (ThermoFisher Scientific) for 30 min at 37°C and 1,000 rpm. Gas chromatography‐mass spectroscopy (GC‐MS) analysis was performed using a Pegasus 4D time‐of‐flight mass spectrometer (LECO) equipped with a Gerstel MPS2 autosampler (Gerstel) and an Agilent 7890A oven (Agilent). The derivatization products were separated on an Rxi‐5Sil^®^ MS column (30 m × 0.25 mm ID × 0.25 μm) (Restek) with an IntegraGuard^®^ pre‐column using ultrapure He at a constant flow of 1 ml/min as carrier gas. The linear thermal gradient started with a 1‐min hold at 50°C, followed by a ramp to 330°C at 20°C min^−1^. The final temperature was held for 5 min prior to returning to initial conditions. Mass spectra were collected at 17 spectra s^−1^. The injection port was held at 250°C, and 2 μl of the sample were injected at an appropriate split ratio. Peak identification was conducted using the Fiehn primary metabolite library (Kind et al., 2009) and the cut‐off threshold of 600 (60%). Peak alignment and spectrum comparisons were carried out using the Statistical Compare feature of the ChromaTOF^®^ software (LECO). The surrogate standard ribitol and the initial nectar volumes were used for normalization. Statistical analyses were performed on normalized data using MetaboAnalyst 3.0 (Xia et al., 2015).

### 2.5 EFN secondary metabolite analysis by GC‐MS‐FID

Chemical standards and extra floral nectar samples were analyzed on an Agilent 7890 A GC System, which was equipped with a DB‐5 column (30 m × 0.2 mm fused silica cap. column, film thickness of 0.25 μm) and operated using the following conditions: injector temp., 240°C; column temp., 60–240°C at 3°C/min, held at 240°C for 5 min; carrier gas, He; injection volume, 5 μl (split on FID, split ratio 25:1); MS mass range from 40 to 650 m/z; filament delay of 3 min; target total ion chromatogram (TIC) of 20,000; a prescan ionization time of 100 μs; an ion trap temperature of 150°C; manifold temperature of 60°C; and a transfer line temperature of 170°C; simultaneous detection with MS and FID by splitting the column outlet (1:1). Detector temperature for FID is 300°C. As a result of GC analysis, three predominant constituents were identified in EFN samples. The compounds were then quantified by performing area percentage calculations based on the total combined FID area. For example, the area for each reported peak was divided by total integrated area from the FID chromatogram from all reported peaks and multiplied by 100 to arrive at a percentage. The percentage is a peak area percentage relative to all other constituents integrated in the FID chromatogram. To verify the chemicals, commercial standards were injected and compared with retention time and mass spectra data of nectar samples. Secondary metabolites were identified by Kovats analysis and comparison of mass spectra with those reported in the NIST mass spectra database. Furfuryl alcohol (Sigma‐Aldrich) and 5‐hydroxymethylfurfural (Sigma‐Aldrich) were also identified by comparison with commercial standards.

## 3 RESULTS AND DISCUSSION

### 3.1 Terpenoid aldehydes (TAs) responses of JACO GL to physical wounding

Upland cotton produces varying amounts of TAs in most parts of the plant including the leaf, boll, seed, boll hull, calyx, and root (Figure 1a) (Scheffler, 2016). The TA biosynthetic pathway is shown in Figure 1b (Benedict, Martin, Liu, Puckhaber, & Magill, 2004). To evaluate the TA level in response to an abiotic stress, physical wounds were made on the third leaf from the bottom of the fully glanded *G. hirsutum* variety JACO GL and after 4 days the wounded leaf was collected along with younger (leaf 4–6) and older leaves (leaf 1–2). Figure 2a shows the production levels of gossypol and other TAs. The top youngest (leaf 6) displayed the highest TA production in both the control and wounded; although, the level decreased as leaves got older. This result is consistent with an earlier study showing that newly emerged leaves exhibit higher terpenoid levels than older leaves (Hagenbucher, Olson, Ruberson, Wackers, & Romeis, 2013). Other reports indicated that terpenoid levels can be further enhanced, up to 15‐fold, after physical damage, herbivory, or JA treatment in comparison to undamaged cotton plants (McAuslane & Alborn, 1998; McAuslane et al., 1997; Opitz, Kunert, & Gershenzon, 2008).

As previously mentioned, gossypol is predominant in seeds while hemigossypolone and heliocides are predominant in foliar glands (Scheffler, 2016). In the leaf samples, (+), (−)‐gossypol levels appeared to be low as expected, ranging from 0.11% to 0.61% (Figure 2a). While the HGQ level was not affected by the physical wounding, the level of heliocides significantly increased up to 1.8‐fold in the younger leaves (leaf 4–6) in response to the physical damage (One‐way ANOVA, *p *<* *0.005). In the older leaves (leaf 1–2), the level of heliocides was increased, but the difference was not statistically significant (*p *<* *0.1). Figure 2b,c, and d show a leaf‐by‐leaf analysis of TA level between control and wounded plants. The heliocide levels in damaged leaves were increased significantly in contrast to the other TAs.

Our results indicate that heliocides can be systemically induced after physical damage, presumably through a signal cascade mediated by phytohormones such as JA and salicylic acid (SA). The wound effects on a single leaf (leaf 3) increased the level of heliocides in the three younger leaves (leaf 4–6) by 1.7–1.8 fold (*p *<* *0.05), but the treatment in this study was not sufficient to increase the production of other TAs (Figure 2).

**Figure 3:**
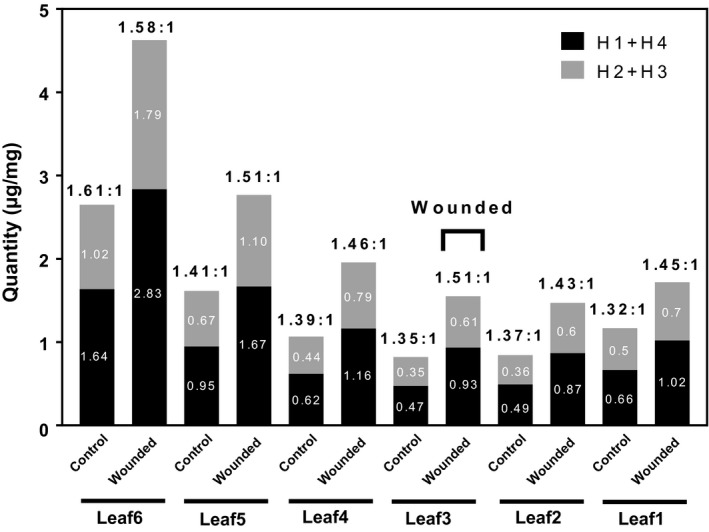
The ratio of foliar H1 + H4 (black) to H2 + H3 (gray) in response to the physical damage. The third leaf was physically wounded for 4 days and leaf samples (leaf 1–6) were collected from control and wounded plants at two time points—Day 1 and Day 5

Heliocides from cotton have been previously identified as natural insecticidal compounds (Stipanovic, Bell, Obrien, & Lukefahr, 1977, 1978a,b). Heliocides are classified into two groups, depending on whether they have a β‐ocimene or myrcene added to the HGQ molecule backbone. Heliocides H1 and H4 have an added β‐ocimene and H2 and H3 a myrcene (Figure 1b). To further investigate if there were any ratio changes between two heliocide groups, the ratio of H1 + H4 to H2 + H3 was compared after the wound treatment. The comparison showed that the plants naturally accumulated more H1 + H4 than H2 + H3, up to a 1.6‐fold difference (Figure 3). The physical damage increased the proportion of H1 + H4 in leaves 1–5, except in the top youngest leaf (leaf 6), which showed a slight decrease. The results indicate that the wound treatment increases the total level of heliocides, particularly the H1 + H4 group, in the younger leaves. Notably, the level of HGQ which is a precursor of heliocide synthesis, was not altered. It may be that HGQ production increased, but was quickly converted to heliocides. It is important to note that this study was performed on one genotype, a *G. hirsutum* variety JACO GL, and that varied TAs accumulation are expected within the same variety, as well as among other genotypes.

### 3.2 EFN production of Deltapine 383 after physical wounding

To determine if the physical wounding stress affected EFN production and its composition, the *G. hirsutum* variety Deltapine 383 that has fully developed extrafloral nectaries was wounded on the third leaf and EFN collected 4 days after treatment.

**Figure 4:**
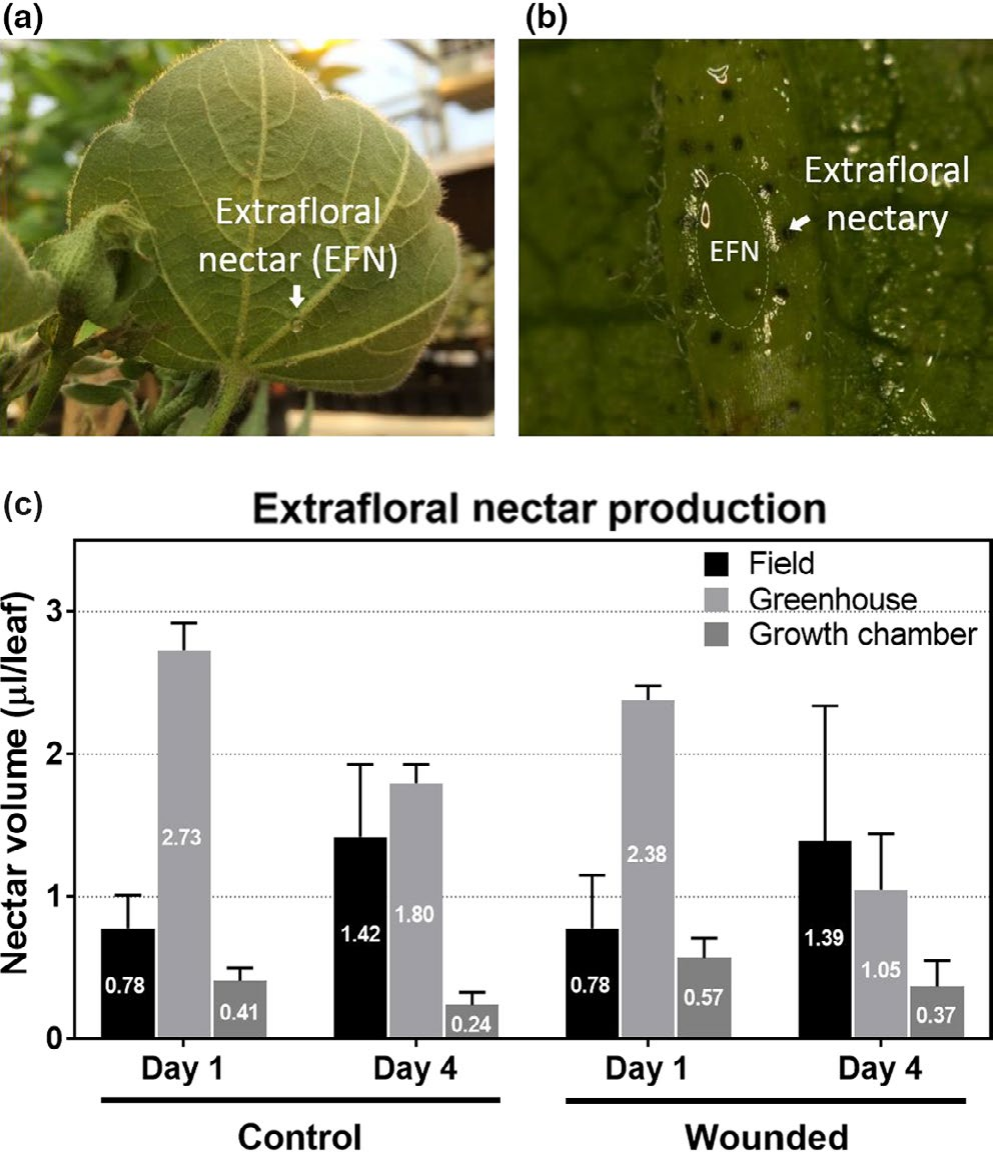
EFN formation in Deltapine 383 (a) in the nectary (b) at the mid‐vein of abaxial leaf. (c) Changes in nectar production in response to wound treatment. The physical wounding was introduced on day 1 and 2. Three biological replicates were used for nectar production estimation. Each replicate includes the nectar collected from 20 plants at day 4. The mean of three replications is displayed with standard deviation

Figure 4 shows the EFN formed in the nectary located on the abaxial side of a leaf on the mid‐vein, and the levels of EFN production under different experimental conditions, including the growth chamber (controlled), greenhouse (semi‐controlled; temperature and day length variation), and field (biotic and abiotic stresses). The different growth conditions enabled us to elucidate how EFN production is correlated with environmental factors. Cotton plants grown in the greenhouse showed the highest pre‐treatment EFN production (2.73 μl/leaf), followed by field (0.78 μl/leaf) and then growth chamber (0.57 μl/leaf). Because of the higher level of natural insect pressure in the field, it was predicted that pre‐treatment field EFN production levels would be the highest. The lower level could be attributed to several environmental factors including high ambient temperature that increased evaporation or insect consumption that decreased the levels in the nectaries. In the greenhouse, EFN production was significantly reduced after the wounding treatment (2.38 to 1.05 μl/leaf, *p *<* *0.05). In the growth chamber, production also decreased, but was not statistically significant (0.57 to 0.37 μl/leaf, *p *<* *0.1). A preliminary experiment in the greenhouse revealed that after 4 days cotton plants were able to replenish their EFN after nectar removal. This indicates that the reduction in volume shown in the greenhouse and growth chamber was not due to the lack of time for nectar formation. Conversely, the field EFN increased by twofold compared to the other locations in both the control and wounding treatment. The increase might be due to rainfall that occurred after wounding, since there were other variables including temperature and humidity that could not be controlled. In summary, the EFN level of Deltapine 383 in the growth chamber and greenhouse decreased after physical damage while the production increased in the field. This indicates that physical damage to a single leaf was not an adequate stimulus to increase EFN production and in this study, the treatment appeared to inhibit production.

### 3.3 Classes of metabolites identified in Deltapine 383 EFN

Cotton EFN contains a wide range of primary metabolites that function mostly as essential factors in cell biosynthetic pathways. The primary metabolites were identified based on comparison of mass spectra to the Fiehn library. Table 1 shows the chemical compounds identified in Deltapine 383 EFN. A total of 78 primary and secondary metabolites were found and categorized into 17 groups. The chemical profiles share compositional similarity with phloem sap, where three main types of sugars exist: sucrose, raffinose‐series oligosaccharides, and polyols (Tarczynski, Byrne, & Miller, 1992). These findings support the theory of nectar's origin that it began as leaky solute from the phloem (De la Barrera & Nobel, 2004), which occurred in developing stems of various cacti and *Eucalyptus* species; however, this should be investigated to further support this theory. The majority of Deltapine 383 EFN metabolites are photosynthates or photosynthetic derivatives produced by other enzymes including cell wall invertases.

**Table 1:** Metabolites identified in upland cotton Deltapine 383 EFN

	Identification	Mass	R.T	Peak area	Counts		Identification	Mass	R.T	Peak area	Counts
1	1,5‐Anhydroglucitol	218	824	26.7	3	40	Lactose 1	204	1,058	3.1	2
2	1‐Kestose	217	1,041	419.6	3	41	Leucrose 1	283	659	0.9	1
3	1‐Monopalmitin	371	844	52.5	3	42	Levoglucosan	159	636	11.0	2
4	2‐Deoxy‐D‐galactose 2	173	919	0.3	3	43	L‐Malic acid	233	523	2.8	3
5	2‐Hydroxypyridine	152	336	0.7	1	44	L‐Threose 1	205	507	0.0	0
6	4‐Hydroxyphenylethanol	179	557	0.0	0	45	Mannose 2	262	667	33.6	3
7	5‐Methoxytryptamine 2	174	399	0.0	0	46	Melezitose	157	1,072	2.4	2
8	β‐Mannosylglycerate 1	204	637	1,142.4	3	47	Melibiose 1	204	880	39.6	1
9	Cellobiose 2	140	740	0.3	1	48	Methyl‐β‐D‐galactopyranoside	204	623	37.8	1
10	Citric acid	273	634	0.0	0	49	Monostearin	57	888	639.2	3
11	Cytidine‐monophosphate degradation product	217	816	68.3	3	50	Myo‐inositol	318	703	14.8	3
12	D‐Altrose 1	201	658	12.6	1	51	Norleucine 2	86	353	0.0	0
13	D‐Arabitol	307	869	139.9	3	52	Octadecanol	327	740	0.6	3
14	D‐Erythro‐sphingosine 2	204	878	0.0	0	53	Oxamic acid	100	358	0.0	0
15	D‐Glucoheptose 1	390	882	11.3	1	54	Palmitic acid	132	709	70.4	3
16	Diglycerol 1	129	806	1.3	2	55	Phosphate	299	441	2.6	2
17	Dihydroxyacetone	70	429	0.0	0	56	Phytosphingosine 2	204	624	0.0	0
18	D‐Talose 2	214	673	0.0	0	57	Prunin degr. Prod. 2	84	1,066	1.1	3
19	Erythrose 2	205	512	0.0	1	58	Raffinose	217	1,079	15.9	2
20	Flavin adenine degradation product	117	576	2.4	2	59	Ribulose‐5‐phosphate 2	315	765	0.6	2
21	Fructose 1	80	653	61.4	3	60	Shikimic acid	220	719	4.1	3
22	Fructose‐6‐phosphate	315	776	0.4	2	61	Sorbitol	277	674	14.0	3
23	Galactinol 3	175	772	3.5	3	62	Sorbose 1	103	625	209.7	1
24	Galactonic acid	292	970	0.2	2	63	Succinic acid	147	458	19.7	3
25	Galactose 1	160	939	1.7	2	64	Sucrose	103	856	24.1	3
26	Gentiobiose 2	437	905	3.0	3	65	Synephrine 1	116	751	0.0	0
27	Glucoheptonic acid 2	217	908	0.0	0	66	Tagatose 1	51	649	1.5	2
28	Gluconic acid 2	361	678	51.6	3	67	Threitol	231	898	4.0	3
29	Gluconic lactone 3	275	672	41.3	2	68	Threonic acid	292	541	0.0	2
30	Glucose 2	105	638	2.8	1	69	Trehalose	129	832	21.2	2
31	Glucose‐1‐phosphate	217	633	18.9	3	70	Tricetin	84	1,067	1.2	3
32	Glycerol	205	437	123.7	3	71	Turanose 1	103	577	0.2	2
33	Glycine 2	100	419	12.5	3	72	Urea	189	428	1.0	3
34	Glycolic acid	66	363	0.6	2	73	Uridine 1	73	1,001	0.0	0
35	Heptadecanoic acid	132	736	0.2	2	74	Xylitol	217	841	0.3	3
36	Hydroxylamine	146	369	0.5	1	75	Xylose 1	291	659	0.0	0
37	Isomaltose 1	160	882	132.6	3	76	Furfuryl alcohol	98	7.4	7,085,594	3
38	Isopropyl‐β‐D‐thiogalactopyranoside	217	1,150	52.6	3	77	5‐hydromethoxyfurfural	126	21.9	50,057,207	3
39	Lactic acid	117	350	30.7	3						

Note

A total of 75 primary (1–75) and two secondary metabolites (76–77) were identified from wounded cotton plants grown at a growth chamber. Primary and secondary metabolites were identified via GC‐MS using the Fiehn primary metabolite library and Kovat's index and the National Institute of Standards and Technology (NIST) database match, respectively. The numbers listed in this table are the average of the mass, retention time (min.), peak area, and number of samples containing the compound of three EFN samples tested. Full EFN metabolic data of cotton plants grown at different growth conditions are listed in a Table S1.

To our knowledge, this is the first report presenting the extended EFN chemical profile of metabolites in addition to carbohydrates, amino acids, and fatty acids. One hypothesis for the origin of EFN is that it may be derived from phloem leaks because of similar chemical compositions; however, nectar contains relatively higher levels of glucose and fructose than phloem solute, which mainly consists of sucrose (De la Barrera & Nobel, 2004). In addition, nectar contains substances generally not found in the phloem sap, such as proteins, organic acids, phenolics and alkaloids (Escalante‐Perez et al., 2012).

### 3.4 Sugars

Our chemical analyses reveal that the EFN contained a variety of mono‐, di‐, and trisaccharides that can readily be used as dietary sources for insects and microbes (Benedict et al., 1981). The sugars we identified included arabinose, galactose, mannose, gentiobiose, lactose, maltose, melibiose, trehalose, melezitose, and raffinose. The simpler sugar products naturally occur in plants and play roles in carbohydrate storage, transport, water deficit tolerance (Patrick, Botha, & Birch, 2013), and pollinator attraction (Johnson & Gregory, 2006). On the other hand, the rarer sugar derivatives are known to be toxic to potential pollinators (Roy, Schmitt, Thomas, & Carter, 2017). In addition to the role as an insect repellent, some sugar derivatives including the disaccharide trehalose and trisaccharide raffinose are known to confer a remarkable capacity to recover from water deficit desiccation (Patrick et al., 2013).

The EFN chemical analyses also identified sugar alcohols (polyols) and sugar acids. Polyols can act as osmo‐protectants against salinity and drought stresses because of their ability to act as a compatible solute (Loescher, 1987; Williamson, Jennings, Guo, Pharr, & Ehrenshaft, 2002), but several studies indicate that polyols retain a much broader role in plant protection based on their antioxidant activity (Williamson et al., 2002). One polyol, erythritol, has been studied for its potential as an organic insecticide; however, they observed detrimential changes in seed germination and growth, suggesting this polyol may have a plant signaling role or be toxic to certain species, such as maize and tomatoes (Scanga et al., 2018).

### 3.5 Flavonoids

Besides the identified sugars, flavonoids were also identified in cotton EFN, including tricetin, a prunin degradation product (naringenin), and 4H‐pyran‐4‐one, 2,3‐dihydro‐3,5‐dihydroxy‐6‐methyl (flavonoid fraction). Flavonoids play a variety of roles ranging from plant growth and development to its interaction with the environments. Furthermore, flavonoids are useful in human health as dietary supplements since they serve as anti‐carcinogenic and anti‐inflammatory agents (Zhou, Gold, Martin, Wollenweber, & Ibrahim, 2006). Plant‐extracted tricin, which is dimethoxylated tricetin, is associated with health benefits in humans due to their antioxidant (Bickoff, Livingston, & Booth, 1964), antiviral (Akuzawa et al., 2011), anticancer (Cai et al., 2004; Hudson, Dinh, Kokubun, Simmonds, & Gescher, 2000), and antihistaminic activities (Kuwabara, Mouri, Otsuka, Kasai, & Yamasaki, 2003).

### 3.6 Amines

Furthermore, two plant amines, 5‐methoxytryptamine and synephrine, were identified in cotton EFN. Biogenic amines are produced by either decarboxylation of amino acids or aldehyde transamination (Bouchereau, Guenot, & Lather, 2000). In plants, amines are actively engaged in a diverse range of cell processes including cell division and differentiation, as well as the biosynthesis of nucleic acids and proteins (Bouchereau et al., 2000). The amine 5‐methoxytryptamine is found in the seeds and fruits of several plant species. It functions as a potent anti‐oxidant, radical scavenger, and radioprotective agent (Badria, 2002). Interestingly, taste contributing amines are found in cotton EFN. For example, synephrine is a plant‐derived “bitter‐taste” amine abundantly found in unripe orange that is often used as herbal medicine (Roman, Betz, & Hildreth, 2007). We speculate that the amine compound may add bitterness to cotton EFN as a counter to the sugar products. High levels of synephrine in EFN may also help to repel the insects.

### 3.7 Nucleotides/Nucleosides

Several nucleotides/nucleosides and their degradation products were also found in EFN. It is known that degraded nucleosides and nucleobases can be recycled to synthesize to new phosphate, nitrogen, and carbon through salvage reactions (Zrenner, Stitt, Sonnewald, & Boldt, 2006). Nucleotides and their degradation products are known as “elicitors sensu stricto*”* since they are compounds that plants use to characterize an initial attack, and induce defense responses to ward of potential predators or pathogens (Heil, 2009). Furthermore, extracellular ATP can act as signaling molecules to lead to diverse range of physiological responses, since it acts as an agonist outside the cell and will not be hydrolyzed (Roux & Steinebrunner, 2007).

### 3.8 EFN secondary metabolites in Deltapine 383 involved in plant defense mechanisms

The GC‐MS coupled with Kovat's retention index analysis and NIST database match enabled identification of EFN secondary metabolites. Furfuryl alcohol and 5‐hydroxymethylfurfural were unequivocally identified by comparison with commercial standards. Table 2 lists secondary metabolites identified in Deltapine 383 EFN with their proposed biological functions. The first identified compound was furfuryl alcohol, which is classified as a furan compound. Previous studies reported that furfuryl alcohol exhibited antioxidant activity, and inhibited microbial proliferation (Chai et al., 2013; Wei, Mura, & Shibamoto, 2001). Another secondary metabolite, 5‐hydroxymethylfurfural (HMF), is known to be heat‐induced, and it is frequently observed in carbohydrate‐rich fruit products when thermally treated (Kowalski, 2013; Zhao et al., 2013). The level of 5‐HMF has been used as an indicator to assess the quality of food products (Khalil, Sulaiman, & Gan, 2010). This metabolite has garnered public interest because of its antioxidant, antimicrobial, and antiproliferative activities (Rosatella, Simeonov, Frade, & Afonso, 2011; Zhao et al., 2013).

**Table 2:** Phyto‐components identified in upland cotton Deltapine 383 EFN by GC‐MS combined with Kovat's retention index analysis and its biological activities

Secondary metabolites	Biological activities	References
Furfuryl alcohol	Anti‐oxidant	Lee, Moon, and Lee (2010) and Wei et al. (2001)
5‐hyrdomethoxyfurfural (HMF)	Anti‐oxidant Antimicrobial Genotoxic	Zhao et al. (2013) Rosatella et al. (2011) Durling, Busk, and Hellman (2009)

In this study, we successfully identified additional secondary metabolites in the upland cotton variety Deltapine 383 EFN. However, due to the limited sample quantity, quantitative analysis was not possible. The chemical profiling conducted suggests that EFN contains secondary metabolites that could provide protective mechanisms against a wide range of biotic and abiotic stresses. Particularly, antimicrobial activity is important to control detrimental microbial populations in the nectary which has been shown to serve as an entry point for a variety of microbes.

### 3.9 Differential EFN metabolic accumulation of Deltapine 383 under different growth conditions

**Figure 5:**
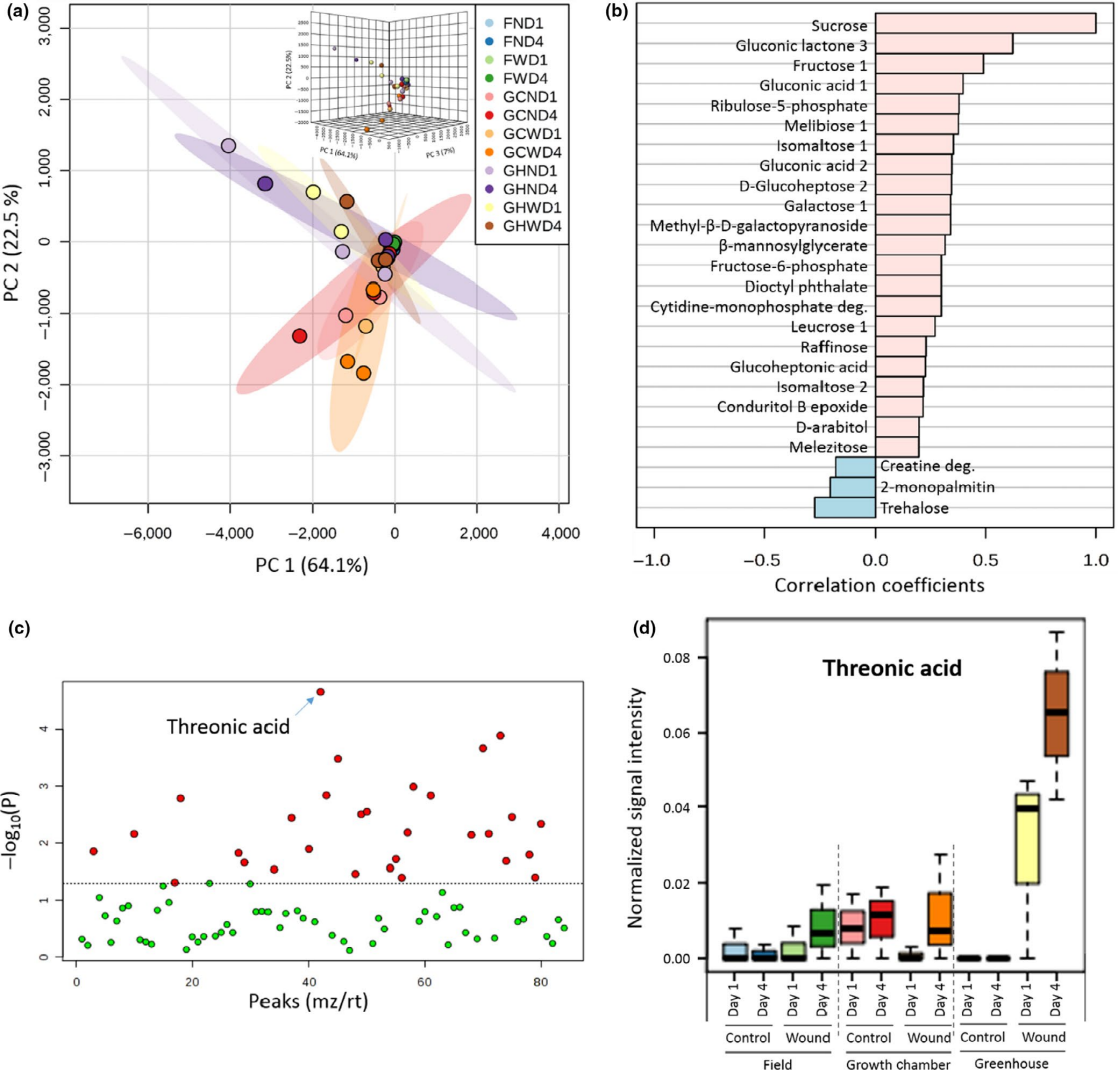
Differential EFN metabolic expression in the Deltapine 383 variety. (a) Principle component analysis (PCA); 2D‐ and 3D score plot of the principle component (PC) 1 and 2 of EFN in the field, growth chamber, and greenhouse. The explained variances are shown on the axis labels in parenthesis. (b) Top 25 metabolites which expression is positively or negatively correlated with sucrose, a known plant stress response compound. (c) A total of 30 metabolites that were significantly changed in Deltapine 383 EFN composition after physical wounding. Red dot indicates the difference is at *p*‐value threshold > 0.05 while green *p *<* *0.05. (d) The compositional changes of threonic acid under field, growth chamber, and greenhouse conditions. Abbreviations; F: Field, GC: Growth Chamber, GH: Greenhouse, N: no treatment, W: wound treatment, D1: Day1, D4: Day 4

Metabolic profiling is an effective and quantitative method to investigate changes of nectar chemicals in response to abiotic stresses (Yu, Du, Xu, & Huang, 2012). To determine whether the physical wounding affected nectar metabolite composition, comparative chemical analyses were conducted using MetaboAnalyst 3.0 (Xia et al., 2015). The analysis reveals that dynamic metabolic reprogramming occurred within 4 days after the wound treatment. Figure 5a is a score plot representing a principle component analysis (PCA) that identifies the directions of two maximum variances in the 80 EFN metabolites. Based on the PCA plot, there were few differences based on a 95% confidence interval in the ellipsoid overlap. However, we identified some metabolites’ accumulation patterns that appeared to vary and were uniquely distributed. To understand the relationship of these metabolites to sucrose, a known compound that increases concentration in response to stress in Arabidopsis, Figure 5b shows the 21 most correlated metabolites to sucrose concentration (Rizhsky et al., 2004). Sucrose is a major photosynthesis product, and it serves as a signaling molecule in systemic carbon redistribution (Chiou & Bush, 1998). Arabidopsis has been reported to increase sucrose concentration in response to abiotic stress (Rizhsky et al., 2004). Of the 30 metabolites that changed, 21 were increased in Deltapine 383 EFN, including sucrose, which supports the stress response in *G. hirsutum* is similar to Arabidopsis. In contrast, 2‐monopalmitin and trehalose levels decreased in response to the treatment.

The heatmap (Figure S1) represents the variable production level of a total of 80 primary and secondary metabolites, of which 30 metabolites significantly changed in response to physical wounding (Figure 5c, One‐Way ANOVA, *p *<* *0.005). Among them, threonic acid appeared to be the metabolite whose production most significantly increased after the treatment under all experimental conditions (Figure 5d). This increase concurs with the previous literature that demonstrated that threonic acid is highly responsive to oxidative stress (Navascues et al., 2012). Heat stress is another one of the major abiotic stresses limiting plant growth and development, which in Arabidopsis, this deficit is shown to correlate to increases in the level of threonic acid (Kaplan et al., 2004). In a study by Levi, Paterson, Cakmak, and Saranga (2011), cotton that was subjected to drought conditions also exhibited increased amounts of threonic acid. For the greenhouse aspect of our study, the physically wounded plants grown under high ambient temperatures (>40°C) exhibited a higher accumulation of threonic acid. It is thought that these increased levels could contribute to plant's capacity to cope with abiotic stresses.

### 3.10 Effect of physical wounding on Deltapine 383 EFN chemical composition

Under greenhouse and field conditions, a direct effect of physical wounding on the Deltapine 383 EFN composition was not clearly evident, possibly due to environmental variation obscuring the changes. For example, in the field, several biotic and abiotic stresses, including additional insect damage, were observed on both the wounded and control plants. Although the greenhouse was controlled for insects, extreme temperatures (>40°C) were recorded during the experiment. In order to minimize environmental variables, an experiment was conducted in a growth chamber to more accurately evaluate the effect of direct physical wounding on EFN composition.

**Figure 6:**
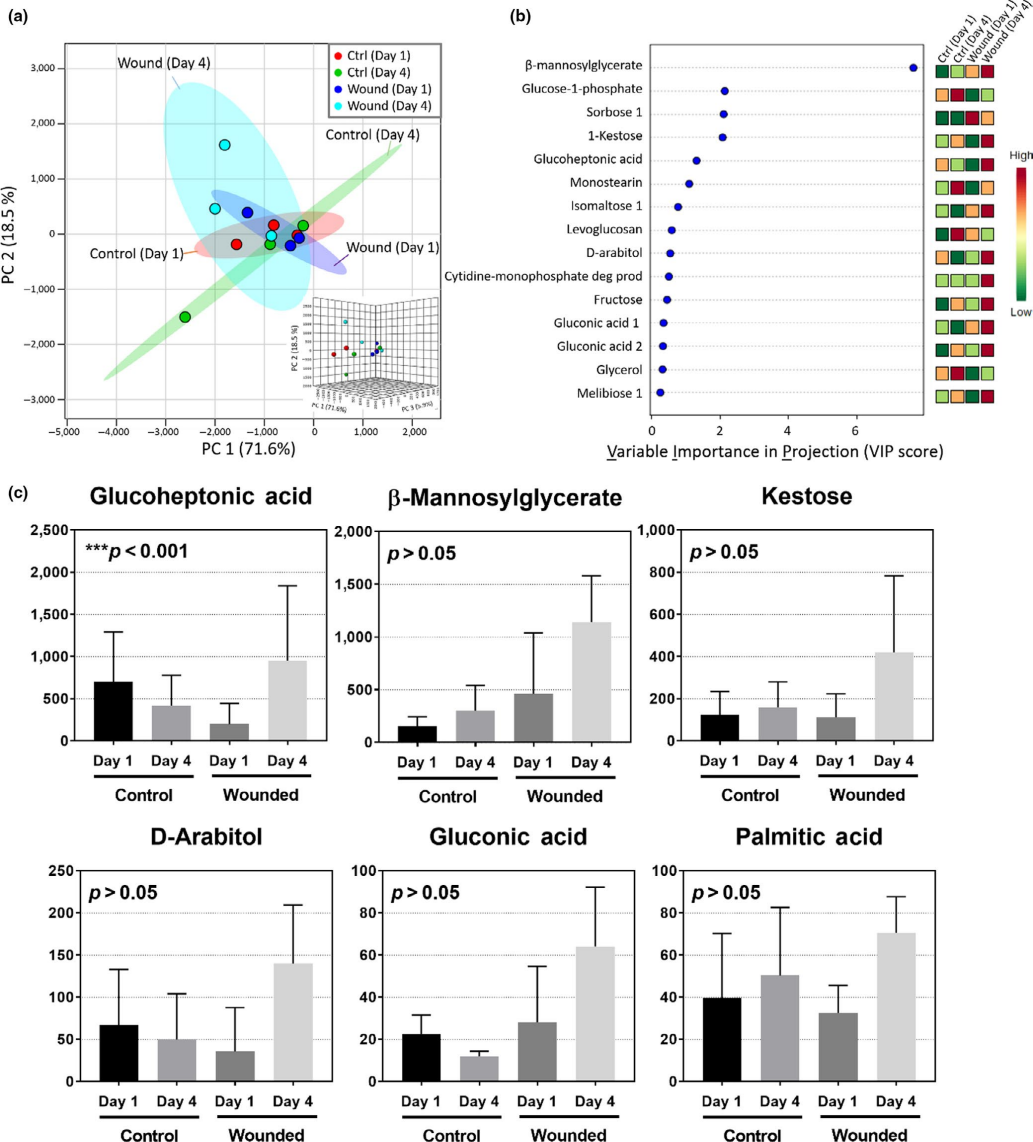
The Deltapine 383 EFN metabolic changes in the growth chamber in response to physical wounding. (a) Principle component analysis (PCA). (b) Variable Importance in Projection (VIP); the colored boxes on the right indicate the relative concentrations of the corresponding metabolite under growth chamber conditions. (c) Top six metabolites that were changed after wounding (One‐way ANOVA and Fisher's LSD as a post hoc analysis)

In Figure 6a, the PCA plot shows that the EFN metabolic profile of Deltapine 383, sampled from the growth chamber, differed after physical wounding. Figure 6b shows the Variable Importance in Projection (VIP score) that represents the importance of the individual variables in each dimension of the multivariate analysis method regardless of the treatment and control groups, such as partial least squares discriminant analysis (PLS‐DA) (Xia, Psychogios, Young, & Wishart, 2009). Based on the VIP score, β‐mannosylglycerate was most significant, followed by glucose‐6‐phosphate, sorbose 1 and 1‐ketose. Figure 6c shows the top six metabolites that significantly changed in Deltapine 383 EFNin response to the wound treatment.

The glucoheptonic acid was significantly over‐produced after wounding (*p *<* *0.001) and five other metabolites β‐mannosylglycerate, kestose, D‐arabitol, gluconic acid, and palmitic acid were also over‐produced, but were not statistically significant (Figure 6c, *p* > 0.05). While little is known about the function of these metabolites, glucoheptonic acid has also been observed as an up‐regulated metabolite in boron‐deficient naval orange plants, which suggests this is involved in stress response (Liu et al., 2015). Furthermore, glucoheptonic acid has been classified as related to the carbohydrate metabolism pathway, which an increase in anabolism of carbohydrates may have led to the observed increase in concentration (Zhao et al., 2015). This is consistent with previous work that investigated energy use related to floral nectar production, suggesting that the physical damage induced nectar biosynthesis may increase carbohydrate metabolism and subsequently increased glucoheptonic acid (De la Barrera & Nobel, 2004).

## 4 CONCLUSION

Over the past 5 decades, cotton TAs have been extensively studied with respect to the cotton plant's defensive mechanisms. Our results show that physical wounding increased the levels of foliar TAs in the fully glanded *G. hirsutum* variety JACO GL, particularly heliocides with insecticidal activity. Prior to investigating cotton EFN's response to physical wounding treatment, we thoroughly profiled EFN of the *G. hirsutum* variety Deltapine 383 that has fully developed extrafloral nectaries to get a baseline profile of the overall metabolic network system. By creating a catalog of metabolites, we can better understand the cotton EFN metabolic composition and gain insights into its ecological functions. Our chemical analyses revealed that Deltapine 383 EFN contains a wide range of primary and secondary metabolites that are involved in cellular metabolism, signal transduction, energy storage, and stress response. Analogous to phloem solutes, our Deltapine 383 EFN profile was composed mainly of carbohydrates, sugar alcohols and acids, carboxyl acids, and lipids with other trace amounts of secondary metabolites involved in defense systems. The composition of Deltapine 383 EFN appeared to vary under different growth conditions, implying that cotton may utilize EFN metabolites to cope with stresses by altering EFN profiles. The strategies include altering the level of antioxidant and antimicrobial secondary metabolites, or by modifying EFN physical properties to a more protective form by increasing viscosity and bitterness.

Although the majority of metabolites remained uncharacterized, these new cotton EFN profiles markedly extend the catalog of known metabolites produced, and provide new insights into the wounding response of cotton plants in terms of the metabolites found in leaf glands and EFN, as well as highlighting some protective functions of secondary metabolites produced in foliar glands and extrafloral nectaries.

## CONFLICT OF INTEREST

The authors declare no conflict of interest.

## AUTHOR CONTRIBUTION

S.H.P., J.S., and B.S. conceived and designed the study; C.L.C performed the chemical analysis; S.H.P conducted the data analysis, interpretation, prepared the manuscript; J.S. and C.S.P edited the manuscript; B.S. funded this research.
