# Supplementary material for: Chemical defense responses of upland cotton, Gossypium hirsutum L. to physical wounding
Source: Plant Direct. 2019 May 17;3(5):e00141. doi: 10.1002/pld3.141 (PMC6589528; doi:10.1002/pld3.141)
Supplement: Supplementary file 1 [file PLD3-3-e00141-s001.docx]

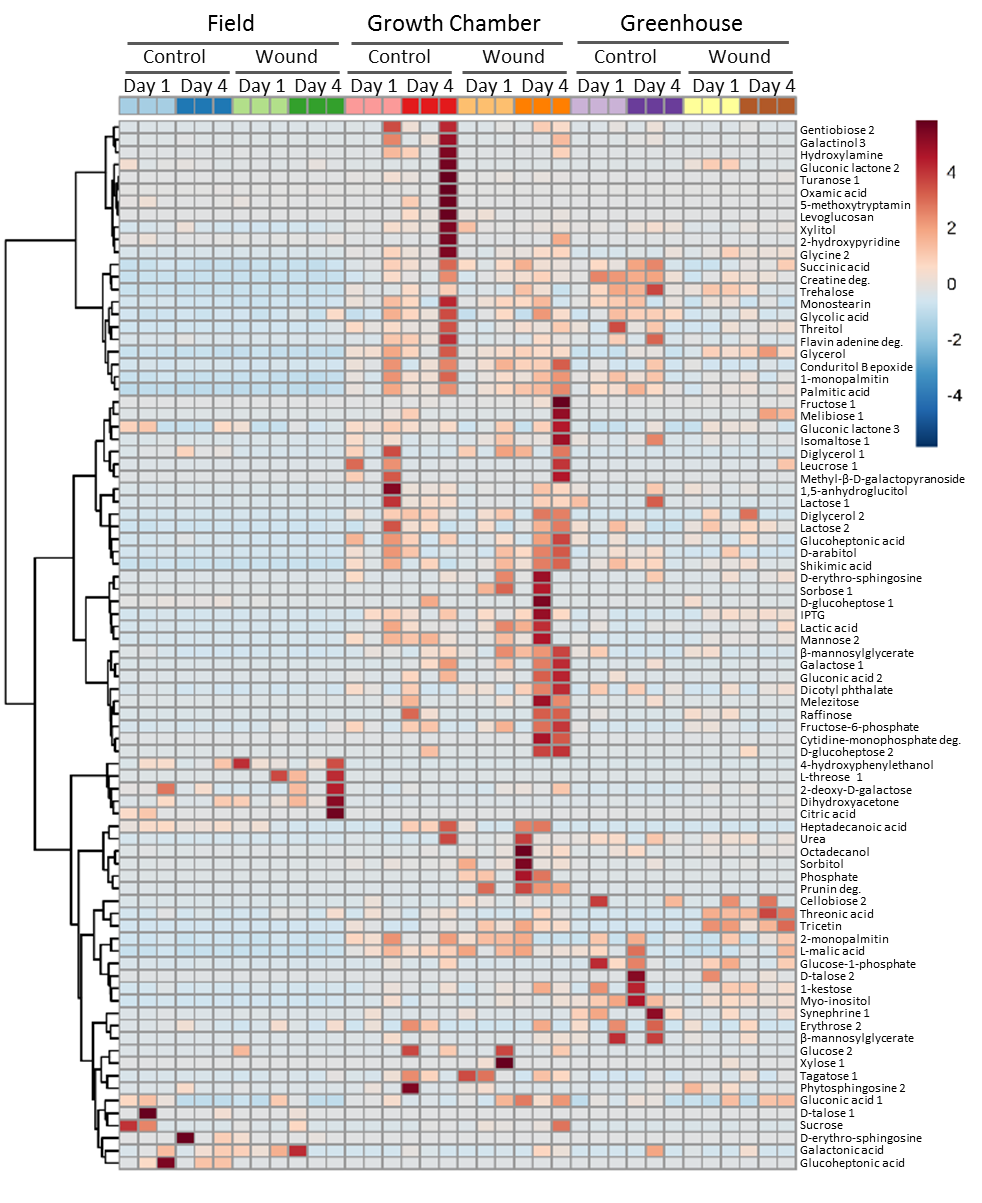


**Supplemental Figure S1**. Heat map displaying clustered Deltapine 383 EFN metabolites and their concentration values which are presented as color scheme. Profiles from each of the three replicates for each condition is shown under each growing location.
