# Supplementary material for: Chemical defense responses of upland cotton, Gossypium hirsutum L. to physical wounding
Source: Plant Direct. 2019 May 17;3(5):e00141. doi: 10.1002/pld3.141 (PMC6589528; doi:10.1002/pld3.141)
Supplement: Supplementary file 3 [file PLD3-3-e00141-s003.docx]

April 24, 2019

Dr. Sang-Hyuck Park
Colorado State University - Pueblo
2200 Bonforte Blvd
Biology Department, LS210
Pueblo, Colorado 81001


RE: Chemical Defense Responses of Upland Cotton, Gossypium hirsutum L. to Physical Wounding

Dear Dr. Park:

Your response to the requested revision has been received. It is understandable that JACO GL and Deltapine 383 were selected for the study of TA and EFN respectively based on the levels of TA and EFN production, if for separate studies. However, it should bear in mind that the response patterns of EFN in JACO GL to treatments might be different from those reported for Deltapine 383, and the response patterns of TA in Deltapine 383 to treatments might be different from those reported for JACO GL. As such, when reporting the results for TA and EFN, you are asked to specify the specific variety throughout the manuscript (Abstract, Results and Discussion). We will be glad to consider your manuscript if this request has been satisfactorily addressed.

As the editor suggested, we have specified the specific variety in the revised version.

Thank you!

To upload your revision, please click the link below.
<https://plantdirect.msubmit.net/cgi-bin/main.plex?el=A2Lr2GK6B1mOj7I3A9ftdKShM47eOGrAQ0vz32E1NQQZ>

In order to provide as timely a service as possible, we ask that your revision is resubmitted within one month after receipt of this request. If an extension is needed, please send a request, along with a brief explanation, to the editorial office at [plantdirect@wiley.com](mailto:plantdirect@wiley.com) .

Please note that, in addition to publishing reviewer comments, the author's responses to review comments will also be published alongside the final version of the paper. If you would not like the author's responses to be published, please contact the editorial office at [plantdirect@wiley.com](mailto:plantdirect@wiley.com) .


Thank you very much for giving us an opportunity to review your work. I look forward to receiving the next version.


Sincerely,

Feng Chen

Editor, Plant Direct
